# CSF Levels of NPTX2 Are Associated With Less Brain Atrophy Over Time in Cognitively Unimpaired Individuals

**DOI:** 10.1002/acn3.70216

**Published:** 2025-10-08

**Authors:** Juan P. Vazquez, Corinne Pettigrew, Yuxin Zhu, Claire Anderson, Guray Erus, Christos Davatzikos, Michael Miller, Abhay Moghekar, Sungtaek Oh, Chan‐Hyun Na, Marilyn Albert, Paul Worley, Anja Soldan

**Affiliations:** ^1^ Department of Neurology Johns Hopkins University School of Medicine Baltimore Maryland USA; ^2^ Department of Biostatistics Johns Hopkins Bloomberg School of Public Health Baltimore Maryland USA; ^3^ Centre for Biomedical Image Computing and Analytics, Perelman School of Medicine University of Pennsylvania Philadelphia Pennsylvania USA; ^4^ Department of Biomedical Engineering Johns Hopkins University Baltimore Maryland USA; ^5^ Institute for Cell Engineering Johns Hopkins University School of Medicine Baltimore Maryland USA; ^6^ Department of Neuroscience Johns Hopkins University School of Medicine Baltimore Maryland USA

**Keywords:** aging, biomarkers, dementia, MRI

## Abstract

**Introduction:**

Neuronal pentraxin 2 (NPTX2) is a synaptic protein involved in synaptic plasticity and regulation of neuronal excitability. Lower baseline cerebrospinal fluid (CSF) NPTX2 levels have been shown to be associated with an earlier onset of mild cognitive impairment (MCI), a pre‐dementia syndrome, even after CSF Alzheimer's Disease (AD) biomarkers (amyloid beta (Aβ_42/40_), and phosphorylated tau (p‐tau_181_)) were considered. To date, however, it is not known whether CSF NPTX2 levels among cognitively unimpaired individuals are associated with longitudinal brain atrophy.

**Objective(s):**

Evaluate the association between baseline CSF NPTX2 levels and measures of long‐term brain atrophy in participants who were cognitively unimpaired at baseline.

**Methods:**

Analyses included 213 participants (*M* baseline age = 57.2 years, 62% female) from the prospective longitudinal BIOCARD study with 13.9 years (max = 22.6 years) of magnetic resonance imaging (MRI) follow‐up, on average. CSF NPTX2 was measured as a composite of three correlated peptides obtained by quantitative parallel reaction monitoring mass spectrometry. MRI brain atrophy was measured longitudinally with three composites. This included two spatial patterns of atrophy: (1) a composite of AD‐signature regions (SPARE‐AD) and (2) a composite of regions sensitive to brain aging (SPARE‐BA), with higher values indicating more atrophy. Additionally, (3) a medial temporal lobe (MTL) composite included volumes of the amygdala, hippocampus, and entorhinal cortex. Linear mixed effect models assessed the association of baseline NPTX2 levels with the rate of change in the brain atrophy measures.

**Results:**

When covarying biomarkers of AD pathology (i.e., the ratio of CSF p‐tau_181_/(Aβ_1‐42_/Aβ_1‐40_), age, sex, APOE4 genetic status, and years of education), lower baseline NPTX2 levels were associated with greater atrophy over time in both AD‐vulnerable regions (SPARE‐AD, standardized estimate = −0.008, *p* = 0.034) as well as regions sensitive to brain aging (SPARE‐BA, standardized estimate = −0.011, *p* = 0.014). These associations were independent of participants having follow‐up diagnoses of MCI or dementia.

**Conclusion:**

Our findings suggest that after accounting for biomarkers of AD pathology, CSF NPTX2 is associated with slower longitudinal atrophy in AD‐signature and aging‐related regions. These findings are consistent with the view that NPTX2 may be a resilience factor in the presence of pathology and modifies rates of neurodegeneration.

## 1 Background

There is substantial interest in better understanding the preclinical stages of Alzheimer's disease (AD), as this may allow for timely interventions that can reduce or prevent irreversible pathology [1]. Of relevance in this regard are markers that may be associated with resilience to AD pathology and AD‐related neurodegeneration, as these could represent novel treatment targets. Current evidence indicates that AD has a prolonged preclinical phase. This phase, which may span decades, is hypothesized to begin with the accumulation of amyloid beta (Aβ) plaques, followed by tau hyperphosphorylation, tau neurofibrillary tangle formation, neurodegeneration, and ultimately cognitive decline [2]. In addition, more abnormal levels of both Aβ and p‐tau, measured in the cerebrospinal fluid (CSF) of cognitively unimpaired individuals, have been linked to accelerated atrophy on magnetic resonance imaging (MRI) scans, particularly in medial‐temporal lobe (MTL) regions [3, 4, 5]. Atrophy during preclinical AD commonly begins in MTL regions, including the entorhinal cortex and hippocampus, and then extends to neocortical areas [6].

Much less is known about synaptic dysfunction and loss during preclinical AD, even though neuropathological studies indicate that cognitive impairment during the symptomatic phase of AD is most strongly linked to synapse damage and loss [6, 7]. This represents an important gap in the literature because the preservation of synapses and synaptic signaling may be an important cellular mechanism of resilience, as suggested by animal models and studies of postmortem human brain tissue [8]. More recent work explored synaptic markers and showed CSF protein levels (including lower CSF NPTX2) associate with higher risk of cognitive decline and AD incidence, further implicating an important role of synaptic dysfunction and suggesting therapeutic target potential for NPTX2 and other synaptic proteins [9]. The current study therefore examind neuronal pentraxin 2 (NPTX2), a protein involved in the regulation of excitatory synapse activity, which has recently emerged as a potential resilience factor in AD and other neurodegenerative diseases [10], and its relationship to brain atrophy.

Evidence from animal models of AD suggests that deletion of the gene encoding NPTX2 increases the detrimental effects of Aβ on inhibitory circuit function [11], a mechanism which may disrupt neuronal circuit activity and lead to impaired synaptic plasticity [12]. It has therefore been hypothesized that lower CSF NPTX2 levels may reflect inhibitory circuit dysfunction and synapse loss [11]. Indeed, NPTX2 levels in CSF decline across the clinical spectrum of AD [13] and lower baseline CSF NPTX2 levels are associated with greater cognitive decline [14], an increased risk of incident mild cognitive impairment (MCI) and dementia [15], and improved prediction of MCI symptom onset after accounting for baseline levels of CSF Aβ_1‐42_/Aβ_1‐40_ and p‐tau_181_ [13].

Few prior studies have examined the relationship between NPTX2 levels and brain atrophy. Prior cross‐sectional studies have reported that lower CSF NPTX2 levels were associated with smaller hippocampal volumes among AD‐dementia patients [11] and with lower cortical thickness in the temporal cortex in a sample that combined participants across the AD spectrum (i.e., cognitively unimpaired, MCI, and dementia) [16]. Similarly, in two short‐term longitudinal studies among participants across the AD spectrum, lower levels of CSF NPTX2 were associated with greater MTL atrophy [16] and more temporal cortex thinning [17] over 2 years. Similar cross‐sectional associations between lower CSF NPTX2 levels and lower cortical thickness or brain volumes have been reported among adults with Down syndrome [18], who have a form of genetically determined early onset AD, as well as among patients with frontotemporal dementia [19, 20]. However, the relationship between NPTX2 levels and brain atrophy in aging and preclinical AD remains unknown.

To address this gap, we examined the association of baseline CSF NPTX2 levels with longitudinal MRI measures of atrophy over 13.9 years, on average (maximum follow‐up = 22.6 years), among cognitively unimpaired middle‐aged and older individuals. Additionally, we examined whether the association between baseline CSF NPTX2 levels and MRI atrophy (using three composite MRI measures) was moderated by CSF AD biomarker levels, and whether it differed among participants who remained cognitively unimpaired compared to those who progressed to MCI or dementia over time. Brain regions of interest included a composite of medial temporal lobe volumes (i.e., entorhinal cortex, hippocampus, amygdala) to capture the earliest AD‐related changes, a global composite of regions associated with AD‐related atrophy (SPARE‐AD index), as well as a composite of regions sensitive to brain aging (SPARE‐BA). We hypothesized that lower baseline CSF NPTX2 levels would be associated with increased atrophy in AD and aging‐susceptible regions, consistent with the view that higher NPTX2 levels may be associated with resilience in both aging and AD.

## 2 Methods

### 2.1 Study Design and Cohort

**FIGURE 1:**
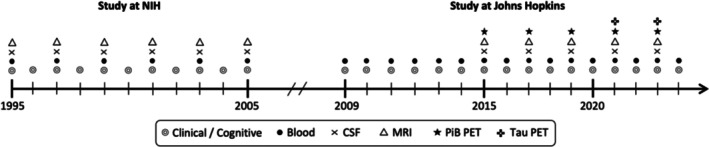
Approximate timeline of the BIOCARD study indicating the types of data collected each year. CSF, cerebrospinal fluid; MRI, magnetic resonance imaging; NIH, National Institutes of Health; PET, positron emission tomography.

Data for this study were derived from the BIOCARD study, the overarching goal of which is to improve our understanding of the preclinical phase of AD and related disorders. The study was started at the National Institutes of Health (NIH) in 1995 and enrolled 349 individuals who were primarily middle‐aged at baseline (mean age = 57 years) and cognitively unimpaired. The study was stopped in 2005 for administrative reasons and re‐initiated at Johns Hopkins University (JHU) in 2009. While the study was at the NIH, participants underwent comprehensive clinical and neuropsychological assessments annually; blood samples, CSF samples, and MRI scans were obtained every 2 years. Since the study has been at JHU, participants have completed detailed annual clinical and neuropsychological assessments and provided annual blood samples. Collection of CSF samples and brain MRI scans was re‐initiated in 2015, occurring approximately every other year. Amyloid positron emission tomography (PET) scans began in 2015, with most participants having their baseline scan between 2015 and 2020; tau PET imaging started in 2021. The PET data were not included in this analysis due to the more limited MRI follow‐up after the baseline PET scans, which precludes modeling of longer term atrophy trajectories. Enrollment of additional participants began in 2020 to increase the cohort size and diversity, but these data are not included in this study. See Figure 1 for a timeline of the study. Participants were included in the analyses if they had at least one CSF NPTX2 measure and valid MRI data on the measures of interest (see below), collected within 15 months of each other while the study was at the NIH (*n* = 214). One participant was excluded from our analyses because they had an estimated age of onset of cognitive impairment prior to their baseline CSF NPTX2 measure, leaving 213 participants in the final analytic sample.

### 2.2 MRI Measures

Brain MRI scans acquired while the study was at the NIH used a standard multimodal protocol on a GE 1.5 T scanner. The scanning protocol included localizer scans, axial Fast Spin Echo sequence (repetition time (TR) = 4250 ms, echo time (TE) = 108 ms, field of view (FOV) = 512 × 512, thickness/gap = 5.0/0.0 mm, flip angle = 90, 28 slices), coronal Spoiled Gradient Echo (SPGR) sequence (TR = 24 ms, TE = 2 ms, FOV = 256 × 256, thickness/gap = 2.0/0.0 mm, flip angle = 20, 124 slices), sagittal SPGR sequence (TR = 24 ms, TE = 3 ms, FOV = 256 × 256, thickness/gap 1.5/0.0 mm, flip angle = 45, 124 slices). Since the study has been at JHU, MRI scans have been acquired on a 3 T MR system (Philips Healthcare, Best, The Netherlands). The protocol included magnetization‐prepared rapid gradient echo (MPRAGE) scans for structural brain imaging (TR = 6.8 ms, TE = 3.1 ms, shot interval 3000 ms, flip angle = 8°, FOV = 240 × 256 mm2, 170 slices with 1 × 1 × 1.2 mm^3^ voxels, and scan duration = 5 min 59 s).

Image harmonization across scanners was achieved using the MUSE software platform [21], as described previously [22, 23]. Briefly, processing of T1‐weighted images included correction of intensity inhomogeneities [24], skull stripping [25], and segmentation of the brain into a set of anatomical regions of interest (ROIs). This method was specifically designed for longitudinal studies to handle differences in scanners and imaging protocols over time and across sites and employs harmonized acquisition‐specific atlases (for details, see [26, 27]). Scanner‐specific atlases share the same ROI labels, imposing consistency of segmentations, while each atlas set preserves the image intensity characteristics of the specific scanner. In this framework, the atlases with reference labels are deformably registered to the participant's native image space, and the candidate labels are fused to generate final ROIs. Accordingly, image processing was performed in each participant's native space. To enhance longitudinal consistency, we employed a pseudo‐4D MUSE strategy in which atlas‐to‐subject deformations for longitudinal scans were constrained to follow a common baseline pathway. Specifically, baseline warps were propagated to follow‐up scans, and label fusion was carried out using these propagated deformations [21]. Preprocessing included N4 bias correction, but no additional steps to correct for movement artifact. This is because the MUSE segmentation method is inherently robust to moderate motion effects by virtue of its multi‐atlas warping and label fusion framework. To further ensure data quality, we conducted systematic visual quality control and excluded cases with evidence of severe motion artifacts. The MUSE pipeline has been extensively validated against benchmark methods and applied in various cross‐sectional and longitudinal studies [21, 27, 28, 29]. MUSE has demonstrated significant improvement in accuracy and more consistent segmentations across scanners, particularly in the segmentation of deep brain structures [30]. The MUSE software package is freely available: https://www.med.upenn.edu/sbia/muse.html. Additional statistical harmonization was applied to the ROI volumes based on the multi‐variate ComBAT‐GAM method [31] to remove protocol‐specific variability. This method simultaneously models scanner effects (unwanted sources of variation) and covariate associations (e.g., age, sex). This harmonization approach integrates a generalized additive model, with a smoothed nonlinear term for age, using thin plate regression splines, and linear terms for sex and intra‐cranial volume (ICV), thereby preserving age and sex differences across sites [31].

A composite score of medial temporal lobe (MTL) volumes was calculated to capture early AD‐related volumetric changes. The composite included harmonized volumes of bilateral amygdala, hippocampus, and entorhinal cortex for each scan. Volumes were first normalized to head size by regressing the bilateral volumes of each region on ICV and saving the standardized residuals from these regressions. The average of the three standardized residuals was calculated to create the composite MTL score, consistent with prior reports [4, 5].

Additionally, we estimated a global pattern of AD‐related atrophy using SPARE‐AD scores, which represent an imaging index of AD‐like neurodegeneration in both medial‐temporal and cortical regions, derived from machine learning, as previously described [32, 33]. To calculate SPARE‐AD scores, a support vector machine classifier with a linear kernel was trained to maximally differentiate between participants without cognitive impairment and individuals with AD dementia, using a dataset of over 10,000 individuals, known as the iSTAGING consortium, which includes the BIOCARD study [27]. More positive SPARE‐AD scores imply a more AD‐like brain structure (i.e., more AD‐related atrophy).

Atrophy associated with brain aging was quantified using SPARE‐BA scores. This MRI approach uses a multivariate pattern regression method based on support vector regression to calculate brain atrophy scores for each participant [27, 34]. The model was trained with T1‐MRI scans, using the harmonized ROI volumes for structures. In the present analyses, SPARE‐BA scores were regressed on age at scan, and the standardized residuals (referred to in the tables as SPARE‐BA‐resid) were used in the analyses presented below, with more positive scores indicating greater age‐related atrophy compared to normative trends. This is comparable to ‘brain age gap’ scores estimated using related techniques [35, 36]. The regions contributing to the SPARE‐BA scores are weighted optimally for estimating age, whereas the regions contributing to SPARE‐AD scores are weighted optimally to distinguish between cognitively normal individuals and individuals with AD dementia.

### 2.3 Cerebrospinal Fluid Biomarker Measures

CSF measures used in the current analyses were derived from samples collected at the NIH (study period: 1995–2005). CSF was obtained via lumbar puncture, after an overnight fast. Immediately after collection, the CSF was aliquoted into polypropylene cryotubes, which were kept on dry ice, and transferred to a −80°C freezer for long‐term storage. Samples were thawed for the first time since collection to measure Aβ_40_, Aβ_42_, p‐tau_181_, and total tau (t‐tau) using fully automated chemiluminescent enzyme immunoassays (Lumipulse G1200 platform; Fujirebio Diagnostics; coefficients of variation obtained from CSF assays are described previously [37]). To summarize levels of AD pathology into a single score, we used the ratio of p‐tau_181_/(Aβ_42_/ Aβ_40_), which combines AD‐specific amyloid β and tau hyperphosphorylation pathology [37, 38]. We used the Aβ_42_/Aβ_40_ ratio instead of Aβ_42_ alone as a measure of amyloid to account for individual differences in total Aβ production and to reduce pre‐analytic variability [39, 40, 41].

CSF samples were thawed for a second time to measure NPTX2 levels using parallel reaction monitoring mass spectroscopy (PRM‐MS) and validated against ELISA, as previously described [13]. Briefly, three separate NPTX2 oligopeptides were synthesized and tagged with stable isotopes on arginine residues, then added to CSF for quantification. Relative peptide abundance was then measured by PRM‐MS. Abundance of the three peptides was highly correlated and therefore combined into a composite score and used as the primary CSF NPTX2 measure for analyses (after *z*‐scoring and averaging) [13]. The CSF NPTX2 composite score rather than the individual peptides was used to reduce the number of analyses, consistent with a previous publication [13]. The CSF NPTX2 composite score was log‐transformed prior to analysis to correct for skewness.

### 2.4 APOE Genotyping

Genotyping was carried out by restriction endonuclease digestion of polymerase chain reaction amplified genomic DNA (Athena Diagnostics, Worcester, Massachusetts). *APOE* ε4 carrier status was coded by a dichotomous indicator variable where ε4 carriers (either heterozygous or homozygous) were coded as 1, and noncarriers as 0.

### 2.5 Statistical Analyses

To compare group differences in participants' characteristics at baseline, t‐tests were used for continuous variables and chi‐square tests for dichotomous variables.

Longitudinal linear mixed effects models were used to estimate the association between baseline CSF NPTX2 levels and level and rate of change in the three MRI measures. Here, baseline was defined as the collection date of the first available CSF NPTX2 measure for which participants also had non‐missing MRI data on the measures of interest obtained within 15 months. If data from more than one MRI scan were available within 15 months of the baseline CSF NPTX2 measure, the MRI data from the scan obtained closest in time to the NPTX2 measure were used for the current analyses. The MRI measures over time were used as the outcome variable, using separate models for each of the three MRI measures. Two sets of primary models were run. Model 1 included the following predictors: baseline NPTX2 composite score, age (at baseline CSF NPTX2 measure), sex, time (in years) since baseline, and the interaction of each predictor with time (i.e., cross product). A sensitivity analysis examined whether results remained the same when also covarying *APOE*‐ε4 genetic status and years of education, as well as their interactions with time. Model 2 was the same as Model 1, but additionally covaried baseline AD pathology levels by including the ratio of CSF p‐tau_181_/(Aβ_42_/Aβ_40_) as an additional predictor, as well as the p‐tau_181_/(Aβ_42_/Aβ_40_) × time interaction term. Terms for *APOE*‐ε4 and the *APOE*‐ε4 × time interaction were also included in Model 2, as *APOE*‐ε4 is known to influence AD pathology levels. In these models, the NPTX2 × time interaction reflects the association between baseline CSF NPTX2 levels with the rate of change in the MRI outcome (slope), while the NPTX2 term reflects the association between baseline CSF NPTX2 with levels of MRI atrophy (intercept).

For both Models 1 and 2, follow‐up analyses examined whether the relationship between baseline CSF NPTX2 levels and MRI atrophy over time differed among individuals who remained cognitively unimpaired compared to those who progressed to MCI or dementia over time. These models were the same as the primary Models 1 and 2 but additionally included terms for follow‐up diagnosis (remained unimpaired vs. progressed), follow‐up diagnosis × time, NPTX2 × follow‐up diagnosis, and follow‐up diagnosis × NPTX2 × time. When the 3‐way interaction was not significant, models were re‐run without this term (and without the NPTX2 × follow‐up diagnosis term) to evaluate whether associations between NPTX2 and brain atrophy remained the same after accounting for progressor status.

In all models, time was modeled in the unit of years (since baseline). A priori statistical significance was set at *p* < 0.05. Data analysis was performed in R, version 4.3.2.

## 3 Results

Baseline demographic characteristics for the 213 participants included in the analyses can be found in Table 1, both for the total sample and separately for participants who remained cognitively unimpaired (*n* = 172) vs. participants who had a diagnosis of MCI or dementia at their last follow‐up visit (*n* = 41, 19.3%, mean time from baseline to onset of symptoms = 8.7 years).

**TABLE 1:** Baseline characteristics of participants.

Variables	Participants
*N*	213
Age at baseline, *M* (SD)	57.2 (10.2)
Female sex, *N* (%)	131 (61.5%)
Years of education, *M* (SD)	17.1 (2.3)
Race, white, *N* (%)	209 (98.1%)
APOE4 Carriers, *N* (%)	73 (34.3%)
Mean AB_42_/AB_40_ ratio, *M* (SD)	0.084 (0.02)
Mean ptau_181_ (SD)	35.32 (17.9)
Mean ptau_181_/ AB_42_/AB_40_ ratio, *M* (SD)	499.9 (505.3)
Composite NPTX2 level, *M* (SD)	0.65 (0.5)
Baseline MMSE score, *M* (SD)	29.7 (0.7)
SPARE‐AD, *M* (SD)	−1.4 (0.9)
SPARE‐BA, *M* (SD)	65.1 (13.8)
MTL composite volume, *M* (SD)	−0.03 (0.83)
Number of MRI measures over time, *M* (SD)	3.8 (1.5)
Years from baseline MRI to last MRI measure, *M* (SD)	12.6 (6.5)
Follow up from baseline NPTX2 to last MRI measure, *M* (SD)	13.9 (5.7)
Number of participants with multiple MRI measures over time	198 (93%)
For participants with multiple MRI measures over time:	
Number of MRI measures over time, *M* (SD)	4.1 (1.4)
Years from baseline MRI to last MRI measure, *M* (SD)	13.6 (5.7)

Results from the first set of models (Model 1) that did not adjust for AD biomarker levels are shown in Table 2. There were no significant associations between baseline CSF NPTX2 levels and level or rate of change in SPARE‐BA or the MTL composite score (all *p ≥* 0.15). For SPARE‐AD, there was a significant association between higher baseline CSF NPTX2 and higher level of SPARE‐AD (*p* = 0.046), but no association with the rate of change in SPARE‐AD over time (*p* = 0.33). Additionally, in these models, older baseline age was associated with higher SPARE‐AD scores, greater increases in SPARE‐AD scores over time, and with greater decreases in MTL volumes over time (all *p* ≤ 0.001). Female sex was associated with lower MTL composite scores at baseline (*p* = 0.011) but not with SPARE‐AD or SPARE‐BA, or with rates of change in the MRI composites. The pattern of results was unchanged when additionally covarying years of education, *APOE*‐ε4 genetic status, and their interactions with time (data not shown).

**TABLE 2:** Results of linear mixed effects analysis for Model 1, showing the relationship between baseline NPTX2 levels and the rate of change in MRI measures.

Variables	MRI measures					
	SPARE‐AD estimate (95% CI)	*p*	SPARE‐BA estimate (95% CI)	*p*	MTL volume estimate (95% CI)	*p*
Age	0.19 (0.1, 0.28)	0.001	−0.06 (−0.179, 0.068)	0.38	0.011 (−0.096, 0.117)	0.85
Sex	−0.026 (−0.24, 0.19)	0.81	0.22 (−0.07, 0.51)	0.13	**−0.32 (−0.57, −0.078)**	**0.011**
NPTX2	**0.11 (0.003, 0.21)**	**0.046**	−0.015 (−0.18, 0.1)	0.60	−0.072 (−0.19, 0.05)	0.25
Time	0.055 (0.044, 0.067)	< 0.001	0.024 (0.012, 0.037)	< 0.001	−0.034 (−0.047, −0.022)	< 0.001
Age × time	0.021 (0.014, 0.029)	< 0.001	0.002 (−0.006, 0.01)	0.62	−0.023 (−0.031, −0.014)	< 0.001
Sex × time	0.002 (0.014, 0.029)	0.94	−0.009 (−0.025, 0.007)	0.25	−0.007 (−0.023, 0.009)	0.40
NPTX2 × time^a^	−0.004 (−0.011, 0.004)	0.33	−0.006 (−0.014, 0.002)	0.15	−0.002 (−0.01, 0.006)	0.57

*Note:* Values in bold denote statistical significance, that is, *p* < 0.05.

^a^ The NPTX2 × time terms for MRI measures reflect the association between lower baseline NPTX2 levels with greater increase in these atrophy measures over time, see text for further detail.

Results from the follow‐up analyses for Model 1 indicated no significant 3‐way interactions between baseline CSF NPTX2, follow‐up diagnosis, and time for the three MRI outcome measures (all *p* > 0.5, data not shown), suggesting that the associations between baseline CSF NPTX2 levels and the rate of change in the MRI measures did not differ for progressors vs. non‐progressors. However, individuals who progressed showed greater increases in SPARE‐AD scores over time (estimate = 0.03, 95% CI (0.012, 0.048), *p* = 0.001) and greater decreases in MTL volume over time (estimate = −0.025, 95% CI (−0.045, −0.005), *p* = 0.015) (see Table S1 for full model results that exclude the nonsignificant 3‐way interactions).

**FIGURE 2:**
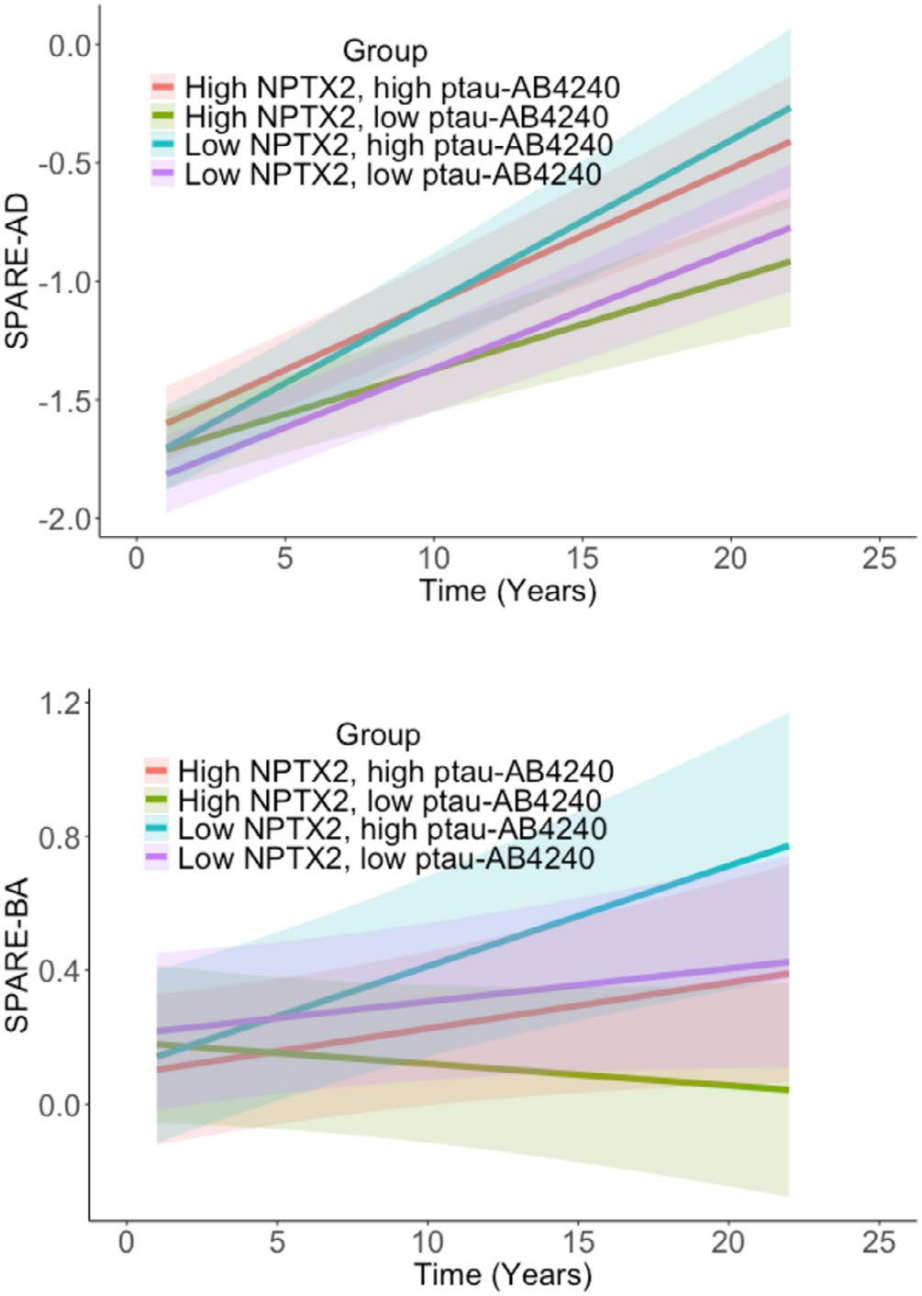
Rate of atrophy over time in SPARE‐AD (top) and SPARE‐BA (bottom) by high versus low levels of baseline CSF NPTX2 and high versus low baseline CSF p‐tau_181_/(AB42/AB40). Higher SPARE‐AD and SPARE‐BA values indicate greater atrophy. Baseline CSF NPTX2 levels and p‐tau_181_/(Aβ_42_/Aβ_40_) ratios were dichotomized by median split for illustration purposes, but treated as continuous variables in all models.

Results for Model 2, which additionally covaried CSF AD biomarker levels (and *APOE*‐ε4 status) are shown in Table 3. Lower baseline CSF NPTX2 composite scores were associated with greater increases in SPARE‐AD (*p* = 0.034; Figure 2, top) and SPARE‐BA (*p* = 0.014; Figure 2, bottom) atrophy measures over time, but not with level or change in the MTL volume composite (both *p* ≥ 0.38). Additionally, in these models, higher p‐tau_181_/(Aβ_42_/Aβ_40_) ratios were associated with greater atrophy over time for all three MRI measures (all *p* ≤ 0.002). Additionally, *APOE*‐ε4 genetic status was associated with greater atrophy in SPARE‐BA regions over time (estimate = 0.017, 95% CI (0.001, 0.033), *p* = 0.04). Similar to Model 1 results, older age was associated with higher SPARE‐AD scores (estimate = 0.16, 95% CI (0.06, 0.256), *p* = 0.002) and greater atrophy over time in SPARE‐AD (estimate = 0.016, 95% CI (0.008, 0.024), *p* < 0.001) and MTL composite scores (estimate = −0.026, 95% CI (−0.035, −0.019), *p* < 0.001). Additional analyses indicated no significant 3‐way interactions of NPTX2 × p‐tau_181_/(Aβ_42_/Aβ_40_) ratios × time for any of the three MRI outcome measures, suggesting similar associations between baseline CSF NPTX2 and rate of change in the atrophy measures for individuals with high vs. low AD biomarker levels at baseline (all *p* > 0.6).

**TABLE 3:** Results of linear mixed effects analysis for Model 2, showing baseline NPTX2 levels in relation to rate of change in MRI MEASURES, accounting for CSF AD‐biomarker levels (ptau:AB42/AB40).

Variables	MRI measures					
	SPARE‐AD estimate (95% CI)	*p*	SPARE‐BA estimate (95% CI)	*p*	MTL volume estimate (95% CI)	*p*
NPTX2	0.08 (−0.028, 0.19)	0.15	−0.015 (−0.16, 0.13)	0.84	−0.039 (−0.16, 0.09)	0.54
ptau:Ab42/AB40	0.097 (−0.007, 0.2)	0.07	−0.093 (0.007, 0.032)	0.19	−0.11 (−0.23, 0.005)	0.062
NPTX2 × time^a^	**−0.008 (−0.016, −0.001)**	**0.034**	**−0.011 (−0.019, 0.002)**	**0.014**	0.004 (−0.004, 0.012)	0.38
ptau:Ab42/AB40 × time	**0.02 (0.009, 0.031)**	**< 0.001**	**0.019 (0.007,0.032)**	**0.002**	**−0.026 (−0.037, −0.015)**	**< 0.001**

*Note:* Values in bold denote statistical significance, that is, *p* < 0.05.

^a^ The NPTX2 × time terms for MRI measures reflect the association between lower baseline NPTX2 levels with greater increase in these atrophy measures over time, see text for further details.

In the Model 2 follow‐up analyses including terms for follow‐up diagnosis (remained unimpaired vs. progressed to MCI or dementia), there were no significant NPTX2 × follow‐up diagnosis × time interactions, suggesting similar associations between baseline CSF NPTX2 levels and atrophy rates among progressors and individuals who remained cognitively unimpaired (all *p* ≥ 0.29). When the non‐significant 3‐way interaction was removed, the association between lower baseline CSF NPTX2 levels and greater increases in SPARE‐BA scores over time remained significant (estimate = −0.01, 95% CI (−0.02, −0.002), *p* = 0.022), while the association between lower baseline CSF NPTX2 and greater rate of change in SPARE‐AD was attenuated (estimate = −0.007, 95% CI (−0.01, 0.001), *p* = 0.077). In these models, as above, higher p‐tau:AB42/40 ratios were associated with greater rates of change in all three MRI measures (all *p* < 0.005), and participants who progressed to MCI or dementia had greater increases in SPARE‐AD atrophy and greater decreases in the MTL composite over time (estimate = 0.026, 95% CI (0.008, 0.044), *p* = 0.005 and estimate = −0.02, 95% CI (−0.039, −0.99), *p* = 0.05, respectively).

## 4 Discussion

In this study, we found that after accounting for baseline CSF AD biomarker levels, lower baseline CSF levels of the synaptic protein NPTX2 were associated with increased long‐term brain atrophy in regions sensitive to both AD and brain aging among individuals who were cognitively unimpaired at baseline. This NPTX2‐related increase in brain atrophy did not differ among individuals who developed cognitive impairment compared to those who remained cognitively unimpaired over an average of 14 years. These findings suggest that NPTX2 levels in mid‐ and later life may be protective against age‐ and AD‐related brain atrophy. Our findings support the view that NPTX2 may be a resilience factor and could be a valuable therapeutic target for decreasing brain atrophy that occurs with aging and in the presence of accumulating AD pathology prior to the emergence of cognitive impairment.

The results of the present study are consistent with and extend prior work. For example, cross‐sectional studies have reported associations between lower CSF NPTX2 and lower brain volumes or cortical thickness among individuals with AD dementia [11], across the AD‐spectrum [17], Down Syndrome [18], and frontotemporal dementia [19, 20]. Two short‐term longitudinal studies among individuals across the AD spectrum also reported a relationship between lower baseline CSF NPTX2 levels and increased atrophy in MTL [16] and AD‐vulnerable temporal [17] regions. This study builds on these prior findings by showing that lower levels of NPTX2 are additionally associated with poorer brain outcomes among cognitively unimpaired participants who have undergone a longer duration of follow‐up than previously assessed.

Interestingly, we did not observe an association between CSF NPTX2 and the rate of change in an MTL volume composite, even though the MTL volumes are included in the SPARE‐AD pattern. Given that the prior short‐term longitudinal studies included participants with MCI or AD‐dementia at baseline, it is possible that lower CSF NPTX2 is more strongly associated with increased atrophy in MTL regions when AD pathology is more advanced. By comparison, among cognitively unimpaired individuals, lower CSF NPTX2 levels may be more strongly associated with increased rates of atrophy in regions outside the MTL that are captured by SPARE‐AD and SPARE‐BA indices, including the insula, cingulate gyrus, prefrontal, and posterior parietal cortical regions, among others [42]. The reasons for these regional differences between different diagnostic groups are currently unclear yet warrant further study.

Previous studies in this cohort have shown that lower CSF NPTX2 is associated with an earlier time to MCI symptom onset [13], and with greater cognitive decline among participants with unimpaired cognition at baseline [43]. A recent longitudinal study looking at different CSF synaptic biomarkers and combining individuals across the AD diagnostic spectrum showed that lower NPTX2 exhibited stronger associations compared to other CSF synaptic biomarkers with incident cognitive decline, and that lower baseline NPTX2 levels were associated with greater amounts of tau aggregation on PET imaging [17]. Unlike other synaptic markers, for which higher values correlated with greater atrophy rates, lower values of CSF NPTX2 were associated with thinning in AD‐vulnerable temporal regions in longitudinal analyses, consistent with what we observed here. These findings, together with ours, may signify that decreased CSF NPTX2 is a sign of synaptic dysfunction, a mechanism associated with brain atrophy. Here, we expand these findings beyond the temporal lobe and to cognitively unimpaired individuals, suggesting these changes may reflect processes underway in the presence of very early pathology. Our findings may also suggest that the relationship between low CSF NPTX2, increased risk of progression to MCI or dementia, and greater cognitive decline may be related to, or mediated by, greater atrophy among individuals with low levels of CSF NPTX2.

Interestingly, in the present analytical models that did not include biomarkers of AD pathology, lower baseline CSF NPTX2 levels were not associated with increased atrophy rates. This may be due to the fact that brain atrophy among cognitively unimpaired middle‐aged and older adults occurs for a variety of reasons, including preclinical AD pathology, as well as cerebrovascular disease [44] and potentially other preclinical pathologies and age‐related processes (e.g., Lewy bodies, TDP‐43, etc.). When statistically accounting for AD‐biomarker levels and their associations with atrophy rates, some of this variability in atrophy is explained, which might make it easier to detect associations of CSF NPTX2 and other variables with atrophy rates. Of note, when AD biomarker levels were not included in the statistical models, lower baseline NPTX2 levels were weakly associated with larger baseline volumes in AD‐signature regions, as measured by higher SPARE‐AD scores. A possible explanation for this seemingly contradictory finding may relate to the fact that individuals with higher baseline CSF NPTX2 levels already had higher AD pathology levels at the study baseline, as suggested by the positive correlation between baseline CSF NPTX2 and p‐tau_181_/AB_42_/_40_ ratios, *r* = 0.25, *p* < 0.01. This could reflect the fact that individuals with higher CSF NPTX2 levels can tolerate higher AD pathology (and more AD‐related atrophy) than individuals with lower CSF NPTX2, while continuing to maintain normal cognition.

In the present study, higher CSF p‐tau_181_/AB_42_/_40_ ratios were also associated with greater atrophy in the aging‐related and AD‐related volumetric patterns, including the MTL volume composite. These results are consistent with many prior cross‐sectional and short‐term longitudinal studies of both cognitively impaired and unimpaired participants that have shown associations between CSF AD pathology markers and atrophy in AD susceptible regions [4, 45, 46]. Extending these prior findings, the present results suggest that AD pathology levels are associated with brain atrophy more broadly during the preclinical phase of AD, possibly reflecting individual differences in the early sites of AD‐related tau accumulation and atrophy, which can include sites outside of the MTL [47, 48].

NPTX2 belongs to a family of neuronal pentraxins along with NPTX1 and NPTXR (neuronal pentraxin receptor). As an immediate‐early gene (IEG), NPTX2 is dynamically expressed by sparse pyramidal neurons in association with learning behaviors and contributes to the homeostatic control of neural activity. NPTX1/2/R are released from presynaptic vesicles into the postsynaptic space in response to neuronal activity and brain‐derived neurotrophic factor (BDNF) [49]. Once in the synaptic cleft, NPTX2 complexes with NPTX1 and binds to NPTXR. This complex can then aggregate AMPA receptors in excitatory synapses on PV interneurons and acts to strengthen the pyramidal neuron drive of inhibition [50, 51]. The NPTX complex also binds and thereby inhibits complement component 1q (C1q) [10], a component of the immune response that is highly upregulated in neurodegenerative diseases, including AD. Since increased complement activity in AD correlates with synapse loss and neurodegeneration [52, 53], elevated NPTX2 levels may thereby protect synapses from microglial engulfment [10]. Taken together, a loss of NPTX2 can therefore drive synapse loss and brain hyperexcitability, leading to neurodegeneration [11, 54].

The concept of resilience in AD refers to mechanisms that protect certain individuals from developing significant cognitive decline despite having a high AD pathology burden. Many mechanisms have been proposed that may explain this discrepancy. On the molecular level, changes in the synaptic proteome have been observed in cognitively normal individuals with AD pathology [55, 56]. Indeed, previous work has shown that higher baseline CSF p‐tau_181_ and t‐tau levels are associated with higher baseline CSF NPTX2 and, inversely, greater rates of CSF NPTX2 decline over time [11, 13]. This initial upregulation and subsequent decline may point to a potentially dynamic process where NPTX2 levels reflect a protective response to AD pathology, followed by declines due to synaptic and/or neuronal loss. Future work using other imaging techniques may help clarify these issues, including diffusion weighted imaging, which may be more sensitive to early neurodegenerative processes and microstructural changes than volumetric measures for both gray and white matter [57, 58, 59].

Our study has several notable strengths, including that the participants in this study were well‐characterized, cognitively unimpaired, and largely middle‐aged at baseline; additionally, they have undergone a long duration of clinical and MRI follow‐up, and have CSF biomarkers available. There are also limitations. Participants in our cohort were predominantly White, highly educated, and have strong family histories of AD. Results may therefore not apply to the wider population. The analyses using three‐way interaction terms to examine whether associations between baseline NPTX2 levels and rate of change in the MRI measures differed by baseline AD biomarker levels or by follow‐up diagnosis may be underpowered given the moderate sample size. We therefore cannot preclude the possibility that the strength of the relationship between lower baseline CSF NPTX2 and increased brain atrophy rates may differ as a function of AD and other pathology levels not measured here. Additionally, this study did not consider how changes in CSF NPTX2 over time impact atrophy rates. Finally, another limitation is that the structural imaging techniques used here lack the spatial and temporal resolution to make causative claims about differences in CSF NPTX2 levels and changes at the synaptic and/or circuit level. As reviewed previously, however, there is strong evidence for NPTX2 as a modulator of structural and functional synaptic plasticity in AD and other neurodegenerative disorders [11, 60, 61]. Cellular and circuit‐level studies may help further increase our understanding of the mechanistic role of NPTX2 in plasticity and resilience against neurodegeneration [62]. Future studies in larger and more diverse samples are needed to replicate these findings and to examine whether other synaptic markers or neurotrophic factors may be associated with similar reductions in brain atrophy. If validated, CSF NPTX2 could serve as a clinically informative biomarker of synaptic resilience, with potential applications in patient risk stratification and therapeutic targeting. Pharmacological interventions aimed at preserving or enhancing NPTX2 expression may also offer new treatment avenues to slow age‐ and AD‐related neurodegeneration and warrant further study.

## 5 Conclusion

In the current study, we examined rates of atrophy in AD‐signature brain regions and regions sensitive to non‐AD‐related aging and their association with CSF NPTX2 levels in individuals who were cognitively unimpaired at baseline. When accounting for biomarkers of AD pathology (i.e., baseline levels of CSF ptau/Aβ42/Aβ40), lower baseline NPTX2 was associated with greater rates of atrophy in both AD‐related and aging‐related regions. Our results provide new insights into NPTX2 as a potentially neuroprotective factor that provides resilience in aging and in response to AD pathology.

## Author Contributions

J.P.V. conceptualization, methodology, formal analysis, writing – original draft writing – review and editing; C.P. conceptualization, methodology, investigation, formal analysis, writing – review and editing; Y.Z. data curation, formal analysis, visualization; C.A. conceptualization, writing – review and editing; G.E. resources, software, writing – review and editing; C.D. resources, software, writing – review and editing; M.M. resources, writing – review and editing; A.M. writing – review and editing, resources, methodology, investigation, data curation; S.O. investigation, writing – review and editing; C.‐H.N. investigation, writing – review and editing; M.A. writing – review and editing, resources, project administration, methodology, funding acquisition, conceptualization; P.W. writing – review and editing, methodology, investigation, data curation, conceptualization; A.S. writing – review and editing, visualization, supervision, resources, methodology, conceptualization.

## Ethics Statement

This study was reviewed and approved by the Johns Hopkins University Institutional Review Board, Study number NA_00027232.

## Consent

Informed consent was obtained from all participants prior to their participation in the study in accordance with the Declaration of Helsinki.

## Conflicts of Interest

The authors declare no conflicts of interest (Juan P. Vazquez, Corinne Pettigrew, Yuxin Zhu, Claire Anderson, Guray Erus, Christos Davatzikos, Michael Miller, Abhay Moghekar, Sungtaek Oh, Chan‐ Hyun Na, Paul Worley, and Anja Soldan). M. Albert is an advisor to Eli Lilly.

## Supporting information


**Table S1:** Results of linear mixed effects models showing NPTX2 levels in relation to rate of change in MRI measures, accounting for follow up diagnosis status (MCI or dementia).

## Data Availability

Data used in these analyses is available through standard application procedures described on the BIOCARD website (biocard‐se.org).
