# Supplementary material for: CSF Levels of NPTX2 Are Associated With Less Brain Atrophy Over Time in Cognitively Unimpaired Individuals
Source: Ann Clin Transl Neurol. 2025 Oct 8;13(2):332–42. doi: 10.1002/acn3.70216 (PMC12883677; doi:10.1002/acn3.70216)
Supplement: Supplementary file 1 — Table S1: Results of linear mixed effects models showing NPTX2 levels in relation to rate of change in MRI measures, accounting for follow up diagnosis status (MCI or dementia). [file ACN3-13-332-s001.docx]

**Supplementary Table 1. Results of linear mixed effects models showing NPTX2 levels in relation to rate of change in MRI measures, accounting for follow up diagnosis status (MCI or dementia).**

|  | ***MRI MEASURES*** | | | | | |
| --- | --- | --- | --- | --- | --- | --- |
| **Variables** | **SPARE-AD Estimate (95% CI)** | **p-value** | **SPARE-BA Estimate (95% CI)** | **p-value** | **MTL Volume Estimate (95% CI)** | **p-value** |
| **NPTX2** | 0.011 (0.004, 0.216) | 0.044 | -0.026 (-0.17, 0.11) | 0.71 | -0.073 (-0.19, 0.05) | 0.25 |
| **Follow-up MCI or Dementia** | 0.078 (-0.1, 0.35) | 0.57 | **0.33 (-0.024, 0.68)** | **0.069** | -0.031 (-0.34, 0.28) | 0.84 |
| **NPTX2 x time*** | -0.003 (-0.01, 0.004) | 0.45 | -0.005 (-0.013, 0.002) | 0.18 | -0.003 (-0.011, 0.005) | 0.45 |
| **Follow-up MCI or Dementia x time** | **0.03 (0.012, 0.048)** | **0.001** | 0.013 (-0.007,0.033) | 0.21 | **-0.025 (-0.045, -0.005)** | **0.015** |

*The NPTX2 x time terms for MRI measures reflect the association between lower baseline NPTX2 levels with greater increase in these atrophy measures over time, see text for further details

Values in bold denote statistical significance, ie. p<0.05
